# Rituximab-Induced Interstitial Lung Disease: A Possible Underestimated Complication—A Systematic Review

**DOI:** 10.3390/cancers17233786

**Published:** 2025-11-26

**Authors:** Alexandra-Simona Zamfir, Mihai-Vasile Marinca, Carmen Lăcrămioara Zamfir, Gabriela Bordeianu, Andrada-Larisa Deac, Bogdan-Mihnea Ciuntu, Cătălina Teodora Pintilie, Doina Ojog, Marcela Brînză, Tudor Andrei Cernomaz

**Affiliations:** 1 Department of Medical Sciences III, Pulmonology, Faculty of Medicine, “Grigore T. Popa” University of Medicine and Pharmacy, 700115 Iasi, Romania; tudor.cernomaz@umfiasi.ro; 2 Department of Oncology, Faculty of Medicine, “Grigore T. Popa” University of Medicine and Pharmacy, 700115 Iasi, Romania; mihai.marinca@umfiasi.ro; 3 Department of Medical Oncology, Regional Institute of Oncology, 700483 Iasi, Romania; 4 Department of Morpho-Functional Sciences I, Faculty of Medicine, “Grigore T. Popa” University of Medicine and Pharmacy, 700115 Iasi, Romania; carmen.zamfir@umfiasi.ro; 5 Department of Morpho-Functional Sciences II, “Grigore T. Popa” University of Medicine and Pharmacy, 700115 Iasi, Romania; gabriela.bordeianu@umfiasi.ro; 6 Department of Oncology, Emergency County Hospital, 400006 Cluj-Napoca, Romania; deac.andrada@umfcluj.ro; 7 Department of Pharmacology, Toxicology and Clinical Pharmacology, Faculty of Medicine, “Iuliu Hațieganu” University of Medicine and Pharmacy, 400012 Cluj-Napoca, Romania; 8 Department of Surgery, Faculty of Medicine, “Grigore T. Popa” University of Medicine and Pharmacy, 700115 Iasi, Romania; bogdan-mihnea.ciuntu@umfiasi.ro; 9 Department of General Surgery, County Clinical Emergency Hospital St. Spiridon, 700111 Iasi, Romania; 10 Department of Otorhinolaryngology, “Grigore T. Popa” University of Medicine and Pharmacy, 700115 Iasi, Romania; catalina.pintilie@umfiasi.ro; 11 Doctoral School, “Grigore T. Popa” University of Medicine and Pharmacy, 700115 Iasi, Romania; ojog.doina@email.umfiasi.ro; 12 Clinical Hospital of Pulmonary Diseases Iași, 700116 Iasi, Romania; marcelabrinza@yahoo.com; 13 Regional Institute of Oncology, 700483 Iasi, Romania

**Keywords:** rituximab, B-cell lymphoproliferative disorders, autoimmune conditions, lung toxicity, interstitial lung disease, immunotherapy

## Abstract

**Background**: Rituximab, a monoclonal antibody targeting CD20, has revolutionized the management of B-cell lymphoproliferative disorders and some immune conditions, significantly improving disease control and patient survival. Beyond its indisputable therapeutic benefits, rituximab can cause serious pulmonary adverse events, particularly interstitial lung disease (R-ILD). Diagnosing R-ILD is challenging due to nonspecific clinical and imagistic features, and its true incidence is possibly underestimated. **Methods**: We conducted a systematic review to synthesize current evidence on R-ILD, focusing on its incidence, diagnostic approaches, management strategies and clinical outcomes. A comprehensive search of PubMed/MEDLINE was performed using the term “rituximab induced interstitial lung disease” through August 2025. Relevant abstracts were screened, and full-text articles meeting the inclusion criteria were analyzed. **Results**: A total of 40 studies were retained after the search and screening, including case reports, case series and cohort studies of R-ILD. This condition was identified in both malignant and autoimmune disorders receiving rituximab, more frequently for combination regimens. Radiological manifestations were heterogeneous, and ground-glass opacities were the dominant pattern. Most R-ILD cases were reversible, but progression to chronic interstitial disease and fatal outcomes are possible. Cohort studies demonstrated variability in incidence, reported instances of successful rituximab reintroduction and suggested a protective effect of prophylactic trimethoprim-sulfamethoxazole against opportunistic pneumonitis. **Conclusions**: Although rare, R-ILD is a clinically significant complication of rituximab therapy. Early recognition and multidisciplinary management are essential, as most patients respond to corticosteroids, while severe cases may progress to respiratory failure or fatal outcomes.

## Simple Summary

Rituximab is an effective therapy option for hematological malignancies and autoimmune diseases; interstitial lung disease is reported among its rarer side effects. Diagnosing this entity may be challenging as clinical and imaging findings are often nonspecific. We conducted a systematic review on rituximab-induced interstitial lung disease published data to provide an overview of its occurrence, clinical presentation, diagnostic features, treatment and outcomes. On a general note, most patients improve after drug discontinuation and corticosteroid therapy, but respiratory failure and death are also possible. By summarizing the existing evidence, this study aims to improve awareness among clinicians and researchers about the importance of early recognition of treatment-related toxicities in the context of chronic disease management, to highlight the role of a multidisciplinary approach and to support the safer and more effective use of rituximab.

## 1. Introduction

Rituximab, a monoclonal antibody targeting CD20, has revolutionized the management of B-cell lymphoproliferative disorders and autoimmune conditions, significantly improving disease control and patient survival [1]. 

**Figure 1:**
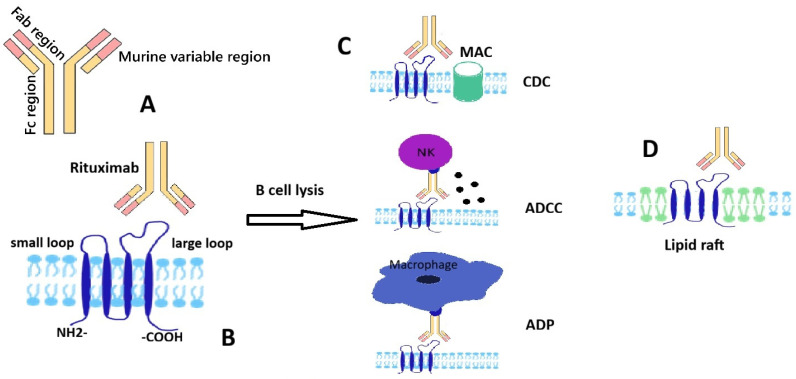
Schematic representation of rituximab and CD20 structures and interaction; main B cell lysis mechanisms. (**A**) A schematic structure of monoclonal antibody-rituximab. (**B**) A schematic structure of transmembrane protein CD20 with two extracellular domains (rituximab epitope located on the large loop). (**C**) Rituximab-induced B cell lysis mechanisms: complement dependent cytotoxicity (CDC) by classical complement cascade leading to formation of membrane attack complex (MAC), antibody dependent cell-mediated toxicity (ADCC) by recruiting natural killer (NK) cells, antibody-dependent phagocytosis by macrophages; (**D**) rituximab-induced redistribution of CD20 to a membrane lipid raft.

CD20 is a tetra-transmembrane protein presenting intracellular N- and C-terminal regions and two extracellular loops which are generally referred to as the small and large loop; these regions are targeted by currently used therapeutic monoclonal antibodies (mAbs) (Figure 1). CD20 presence is strongly associated with the B cell lineage but its absence on stem and plasma cells should be noted. Although used as a target for various immune therapies—next to rituximab, other anti-CD20 agents such as obinutuzumab and ofatumumab are currently in use—its exact physiological interactions and mechanisms are not completely elucidated [2,3]. A potential gatekeeping role for the resting state of the B cell has been advanced for CD20, possibly explained by complex interactions with IgM-class B cell antigen receptor and CD19; along this line, disruption of CD20 seems to potentially promote transformation to plasma cell [4].

Despite its strong link to B cell lineage, there is data describing a CD20 positive T cell subset with distinct properties in terms of migration and adhesivity; this subpopulation seems associated with autoimmune phenomena such as rheumatoid arthritis, psoriasis, multiple sclerosis and possibly some solid tumors [5].

Rituximab is a chimeric immunoglobulin containing murine variable regions and human kappa and Fc sequences. The presence of a variable murine region (Fab) and a human Fc region in the structure of mAb Rituximab (Figure 1) ensures a stronger immune response due to the better binding of the Fc region with human immune effectors. The most studied mechanisms by which the binding of Rituximab to transmembrane protein-CD20 leads to cell lysis are antibody-dependent cell-mediated cytotoxicity (ADCC), complement-dependent cytotoxicity (CDC) mediated by the classical pathway of the complement system and direct apoptosis signaling (Figure 1). For ADCC, the binding of the variable region of Rituximab to CD20 facilitates the binding of its Fc region to FcγRIII receptors on NK cells followed by a cytotoxic response—the release of perforins and granzyme [6]. Granzyme B enters cells through the pores created by perforins, triggering programmed cell death via the caspase pathway [7]. Rituximab is a type 1 anti CD20 antibody and has the effect of redistributing the protein into lipid rafts with potential apoptotic consequences; similarly to other type 1 agents, the ability to activate the complement cascade seems particularly important in explaining its biological effects; crosslinking by FcR was also considered as a putative cytotoxic mechanism at least in vitro [8]. 

Despite its role in the management of lymphomas and potentially some dysimmune conditions, rituximab use is associated with a range of adverse effects, either infusion-related or taking time to develop, including allergic reactions, various blood disorders (possibly severe lymphopenia and myelosuppression), cardiovascular effects (mainly arrhythmias and possibly acute coronary syndromes), tumor lysis syndromes and secondary infections (potentially leading to sepsis). Among these, respiratory complications and particularly the development of interstitial lung disease (ILD) stand out as rare, but potentially life-threatening events. Their uncommon occurrence, coupled with the diagnostic challenges in accurately identifying the underlying pathology, makes these complications particularly difficult to recognize [9]. Although the true incidence of rituximab-induced ILD (R-ILD) remains uncertain, it is likely underestimated due to the nonspecific nature of its respiratory manifestations [10]. In some cases, patients may remain asymptomatic, with ILD detected incidentally during routine imaging follow-up of the underlying condition [11]; the most common computed tomography findings are ground glass opacities (GGOs), frequently accompanied by areas of consolidations, centrilobular nodules or pleural effusion. Additional patterns may include alveolitis, alveolar hemorrhage, and, in chronic cases, pulmonary fibrosis. In the diagnostic process of R-ILD, bronchoscopy with bronchoalveolar lavage (BAL) is primarily used to exclude alternative conditions, whereas lung biopsy provides greater value by defining the histopathological pattern of the lesion. Once the diagnosis is established, management should focus on treating the pulmonary condition, with a multidisciplinary approach to determine whether rituximab therapy can be safely continued [12]. R-ILD is typically managed by interrupting rituximab, corticosteroids and providing supportive care (oxygen therapy, non-invasive ventilation, empirical antibiotics, *Pneumocystis jirovecii* prophylaxis, etc.). Such approaches may lead to complete recovery but progression to respiratory failure and even fatal complications are possible. This highlights the need to recognize R-ILD as a potential adverse event and ensure its prompt diagnosis [11,12,13].

This review aims to synthesize current available data to assess the impact rituximab has in the development of interstitial lung disease, focusing on its incidence, diagnosis, management and outcomes. In addition, particular attention is given to differential diagnosis from opportunistic infections, the radiological and pathological patterns that may occur, and the prognostic implications for affected patients, stressing the importance of timely recognition and clinical outcomes.

## 2. Materials and Methods

**Figure 2:**
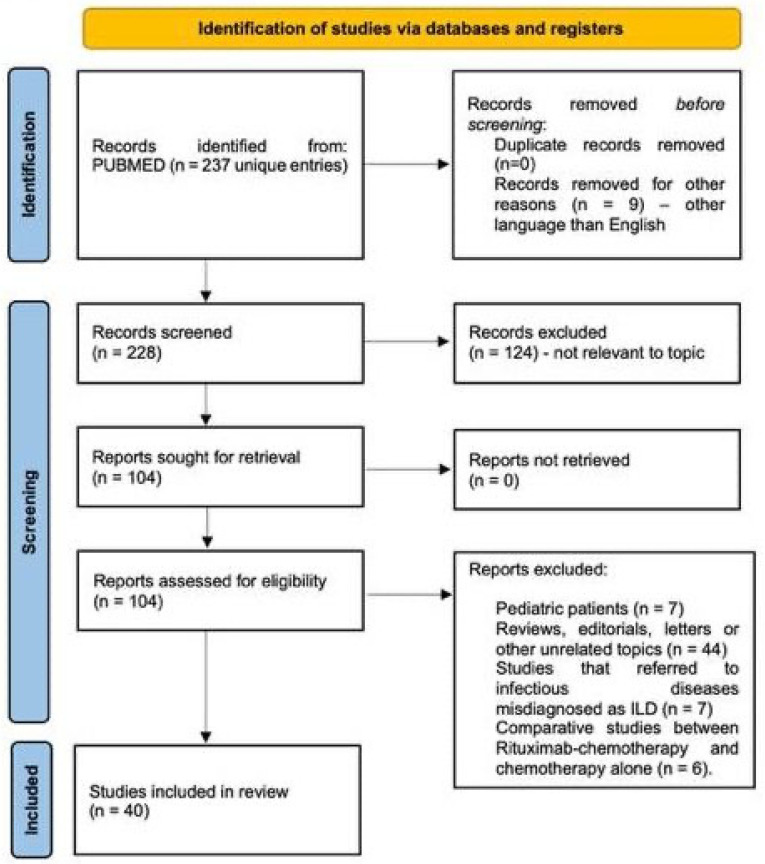
PRISMA flowchart illustrating the article-selection process. Out of 237 potential studies from Pubmed/MEDLINE, 40 papers were included in the final analysis.

We conducted a systematic search of the PubMed/MEDLINE database using the keywords “Rituximab induced interstitial lung disease”, with no time restrictions, up to August 2025. The primary objective was to identify and analyze published material relevant to the development of interstitial lung diseases following rituximab administration. The initial search identified 237 articles; all abstracts were screened to assess their relevance to the topic using the following inclusion criteria: cohort, case series or case presentation of R-ILD occurring within adult populations. When abstracts were considered adequate by at least two reviewers, the full text article was retrieved, explored in detail and included in the final analysis. The overall selection process is illustrated in the PRISMA flow diagram (Figure 2 and Supplementary File S1). A primary data table was generated containing the following parameters in a free form format: author, title, publishing year, type of article, number of patients (with R-ILD and total number of patients for cohorts), disease (specific entity and malignant/non-malignant category), therapy (rituximab-containing regimen, other concomitant medication), time to ILD onset (number of therapy cycles, time from last infusion of rituximab), type of ILD (imagistics, pathology), functional impact, treatment, and outcome. Case series were split into individual cases whenever there was enough information to do so; the table containing individual cases was used to analyze gender ratios and time to onset of R-ILD. Cohorts containing rituximab-treated patients were pooled together to estimate global incidence of R-ILD. Some data such as computed tomography, treatment or outcome was parsed but no attempt to standardize data was made given the high variability of expression and the risk to introduce bias.

## 3. Results

A total of 40 studies were considered after the search and screening process concluded. Core details and patient outcomes are outlined in Table 1.

Across the analyzed reports, rituximab was most frequently administered as part of combination chemo-immunotherapy regimens for lymphomas, the prototypical example being R-CHOP (rituximab, cyclophosphamide, doxorubicin, vincristine, prednisone), considered a standard of care. Variations in CHOP were also observed, including R-CDOP (with liposomal doxorubicin), R-CEOP (with etoposide replacing doxorubicin), R-CVP (without doxorubicin), and R-ACVBP. In addition to these regimens, rituximab was combined with bendamustine (R-Bendamustine), frequently followed by maintenance rituximab, and in some settings with fludarabine- or cyclophosphamide-based regimens (such as R-MAD or fludarabine–cyclophosphamide combinations). More intensive protocols were also reported, including DA-EPOCH-R. Rituximab was occasionally administered with glucocorticoids alone or together with plasmapheresis, cyclosporine, angiotensin-converting enzyme inhibitors (ACE) inhibitors or trimethoprim–sulfamethoxazole (TMP-SMX) prophylaxis, particularly in autoimmune or immune-mediated diseases. In other instances, it was used as monotherapy, either in induction or long-term maintenance schedules.

Our research includes 30 case reports, which identified R-ILD in 16 men and 14 women. While the majority of patients in these studies were diagnosed with B-cell non-Hodgkin lymphomas (NHLs), a subset presented with other conditions, including neurological disorders (aquaporin-4-positive neuromyelitis optica spectrum disorder), hematologic autoimmune disease (immune thrombocytopenic purpura), dermatologic disease (pemphigus vulgaris), renal involvement (fibrillary glomerulonephritis, lupus nephritis with thrombotic microangiopathy) and systemic autoimmune disorders (systemic sclerosis, ANCA-associated vasculitis, primary Sjögren’s syndrome). Time to ILD manifestation after rituximab exposure varied considerably across reported cases, with no predictable temporal association. Among the reported cases, ILD was reversible in 22 patients (twelve women, ten men), chronic in 4 patients (two women, two men) and fatal in 4 patients (one woman, three men).

The case series generally reported small cohorts ranging from two to nine patients, with most studies noting a predominance of male subjects. Nevertheless, the radiological manifestations in these patients were highly heterogeneous, ranging from interstitial pneumonitis, hypersensitivity pneumonitis and ILD patterns such as nonspecific interstitial pneumonia (NSIP) and usual interstitial pneumonia (UIP), as well as advanced fibrotic changes.

A total of five cohort studies were focused on the development of ILDs during treatment with rituximab and chemotherapy in patients with NHLs. One of the biggest studies comprised more than 300 patients (the majority being males) and highlighted several important aspects [14]. In this study, the predominant lung injury pattern was GGO, a finding that was consistent with most of the other reports. On follow-up imaging, the majority of patients demonstrated resolution or improvement of ILD. A smaller proportion required mechanical ventilation, while those with concomitant infections were managed with combination antimicrobial therapy. In most cases, rituximab was successfully reintroduced without recurrence of pulmonary toxicity. On the other side, another study comprising 38 patients revealed that only in five patients Rituximab administration was continued after the diagnosis of R-ILD [15]. 

**Table 1:** Publications on rituximab-associated interstitial lung disease (n = 40), including case reports, case series and cohort studies. References were retrieved through PubMed/MEDLINE and are presented in the order generated by the search engine output. Key clinical features and patient outcomes are summarized.

First Author	Year Published	Disease	Cohort Size (n = 1 for Case reports)	Gender (M/F)	Age (years)	Therapeutic Regimen and Concomitant Therapy	Therapy Courses Before ILD Onset	Time to the ILD Onset Following Last Rituximab Infusion	ILD Radiological Pattern/Pathological Pattern	Severity (Reversible/Chronic/Fatal)	Management (Drug Discontinuation, Corticosteroids, Supportive Therapy) and Follow up
Chari, R. et al. [12]	2025	DLBCL	1	F	73	R-CHOP	1	14 days	Multifocal GGO in all 5 lobes, emphysema, and trace left pleural effusion with atelectasis	Reversible	R-CHOP was maintained, accompanied by an intensified corticosteroid schedule (prednisone 100 mg for 5 days, followed by a 40 mg maintenance dose) and *Pneumocystis jirovecii* prophylaxis, with four additional cycles planned to complete the chemotherapy regimen
Park, S.Y. et al. [15]	2017	DLBCL (n = 33) Follicular lymphoma (n = 3) Mantle cell lymphoma (n = 2)	38	M-25 F-13	63 ± 12	R-CHOP	4 (median value)	-	Diffuse GGO followed by patch GGO, multifocal airspace or alveolar, diffuse airspace or alveolar, and diffuse reticular pattern	Reversible; one fatality	5 patients continued the treatment with rituximab
Aagre, S. et al. [16]	2015	Stage IIIB follicular lymphoma	1	F	33	R-CHOP	4		Bilateral patchy GGO	Reversible	Severe hypoxemia was managed with IV methylprednisolone followed by oral taper. Rituximab was discontinued; CHOP was given without further lung injury.
Zou, W. et al. [14]	2024	DLBCL (n = 256) Marginal zone lymphoma (n = 35) Follicular lymphoma (n = 8)	321	M-189; F-132	48	R-CHOP	-	1.7 months	GGO in 289 (90.0%) patients; 118 (37.1%) patients presented reticulations; 72 (22.4%) patients had centrilobular nodules; 49 (15.3%) patients presented consolidation; and 28 (8.7%) patients were affected by traction bronchiectasis.	269 patients (83.8%) demonstrated improvement on HRCT	Glucocorticoids were used in 196 (61.1%) subjects. 35 (10.9%) patients required mechanical ventilation. 281 patients (87.5%) restarted Rituximab therapy.
		Burkitt lymphoma (n = 7) Mantle cell lymphoma (n = 7) High-grade B-cell lymphoma (n = 5) Chronic lymphocytic leukemia/small lymphocytic lymphoma (n = 3)									
Naqibullah, M. et al. [17]	2015	1. DLBCL 2. Stage IV follicular lymphoma 3. Mantle-cell lymphoma 4. Large B-cell lymphoma 5. Mantle-cell lymphoma	5	M-5	1. 68 2. 71 3. 70 4. 63 5. 63	1. R-CHOP 2. R-Bendamustine 3. R-CHOP 4. R-CHOP plus Rituximab monotherapy 5. R-Bendamustine	1. 3 2. 2 3. 2 4. 3 R-CHOP followed by 3 Rituximab	1. 5 months 2. 1 month 3. 1 week 4. Unspecified 5. Unspecified	1. GGO and fine sub-pleural reticulation with sparing of the immediate sub-pleural lung, compatible with NSIP. 2. Diffuse fibrotic interstitial pneumonitis, with bilateral patchy consolidation and GGO, intervening with a normal lung. 3. Diffuse GGO with areas of consolidation. 4. Extensive partly confluent small nodules in a centrilobular pattern with characteristic sparing of the subpleural region, and mosaic attenuation, suggestive for hypersensitivity pneumonitis. 5. GGO and nodular changes.	1. Reversible 2. Chronic 3. Fatal 4. Chronic 5. Reversible	Rituximab discontinued Rituximab discontinued; corticoteroids improved dyspnea and DLco (50%); HRCT showed GGO regression with persistent reticulation. Intensive care *P. jirovecii* therapy Rituximab discontinued; prednisolone improved symptoms and DLco, relapse occurred on tapering. Prednisolone
Mahmoud, M. et al. [18]	2022	Stage 4 mantle cell lymphoma	1	M	73	R-Bendamustine	6	Three weeks	GGO and septal thickening	Fatal	Antibiotics, corticosteroids; the patient was transferred to long-term care.
Kong, H. et al. [19]	2014	Idiopathic thrombocytopenic purpura (ITP)	1	M	30	Rituximab monotherapy	4	Three weeks	Diffuse bilateral GGO; patchy consolidation of both lower lobes	Reversible	Steroid therapy led to marked improvement; after a 4-week taper, 1-month CT confirmed complete resolution of infiltrates.
Ahn, S.H. et al. [20]	2018	Aquaporin-4 immunoglobulin G-positive neuromyelitis optica spectrum disorder (NMOSD-AQP4)	1	F	48	Rituximab monotherapy	4		Patchy GGO accompanied by subsegmental linear atelectasis on the bilateral lower lobes	Reversible	Rituximab cessation alone; CT and pulmonary function tests were normal after 8 months.
Child, N. et al. [21]	2012	Immune thrombocytopenia purpura (ITP)	1	M	84	Rituximab monotherapy	3	One week	GGO consistent with active pneumonitis associated with developing fibrosis	Chronic	Corticosteroids; patients developed progressive dyspnea; HRCT showed worsening diffuse fibrosis.
Kalyankar, P.P. et al. [22]	2025	Pemphigus vulgaris (PV)	1	F	42	Corticosteroids and rituximab	2	One month	Multiple GGOs in bilateral lung fields with basal subsegmental atelectasis and pleural thickening	Reversible	Pulse methylprednisolone therapy.
Sainz-Prestel, V. et al. [23]	2013	Fibrillary glomerulonephritis	1	M	49	Corticosteroids and rituximab	2	Five weeks	Bilateral interstitial pulmonary infiltrates without pleural effusion	Reversible	IV antibiotics and high-dose steroids; discharged from ICU after 10 days.
Zayen, A. et al. [24]	2011	Stage III diffuse large B cell lymphoma	1	F	29	R-CHOP	8		Diffuse bilateral lung infiltrates with macro- and micronodular densities	Reversible	Corticosteroids; complete resolution of the interstitial disease after 2 months.
Albusoul, L. et al. [25]	2023	Multiple myeloma and follicular B-cell non-Hodgkin lymphoma	1	M	55	R-bendamustine and rituximab monotherapy	6 + 5		Basal patchy airspace disease and ground-glass opacities suggestive of multifocal pneumonia or drug-induced pneumonitis	Reversible	Rituximab discontinuation; corticosteroids; TMP-SMX for *Pneumocystis jirovecii* prophylaxis.
Wu, Y. et al. [26]	2013	DLBCL	1	F	71	R-CHOP	3	3 weeks	Bilateral generalized GGOs in both lungs.	Fatal	Intensive steroid treatment and etanercept with progression to fatal respiratory failure
Tonelli, A.R. et al. [27]	2009	Chronic lymphocytic leukemia.	1	F	59	Corticosteroids and rituximab	4	3.5 weeks	Bilateral GGOs with centrilobular nodules and mosaic attenuation, compatible with hypersensitivity pneumonitis.	Reversible	Corticosteroids with clinical response within several days.
Ergin, A.B. et al. [28]	2012	Recurrent nodal marginal zone B-cell stage 4 lymphoma	1	M	82	Rituximab monotherapy	1	4 days	Extensive bilateral patchy infiltrates affecting most of the lung parenchyma; biopsy revealed fibroblastic proliferation consistent with BOOP, without evidence of malignancy or atypia.	Reversible	Corticosteroids, with functional improvement.
Subramanian, M. et al. [29]	2010	DLBCL	1	M	53	R-CHOP	5	1 month	Diffuse GGO, suggestive for subacute interstitial pneumonitis	Reversible	Prednisolone was tapered over 2 weeks; subsequent CHOP and radiotherapy controlled NHL. On follow-up, the patient remains asymptomatic for both NHL and ILD.
Park, G.H. et al. [30]	2010	Primary Cutaneous Intravascular Large B-cell Lymphoma (IVLBL)	1	F	62	R-CHOP	3		New bilateral diffuse ground-glass opacities, predominantly peripheral in the upper lobes, with a small left pleural effusion. Biopsy revealed alveolar pneumocyte hyperplasia and intra-alveolar hyaline membrane formation.	Reversible	Rituximab discontinued Corticosteroid treatment CHOP was readministered 6 weeks after the discharge.
Leon, R.J. et al. [31]	2004	Follicular cell non-Hodgkin lymphoma	1	M	56	Rituximab monotherapy	12	Three weeks	Diffuse pulmonary infiltrates extending from the hilum to the periphery, most marked at the lung bases. Biopsy demonstrated atelectasis, bronchiectasis, extensive interstitial fibrosis with focal chronic inflammation, organizing changes, and arterial thrombosis—consistent with acute pulmonary fibrosis.	Chronic	Intravenous antibiotics, high-dose corticosteroids and oxygen therapy.
Wei, W. et al. [32]	2021	DLBCL	25/556	M-25	53 (mean age)/55 (mean age)	R-CHOP/ R-CDOP	3.3 (median value)	16.4 ± 2.7 days from the last administration	All patients presented diffuse GGOs	Fatal (respiratory failure one case); reversible	Corticosteroids; antibiotics for patients with infections.
Kim, K.M. et al. [33]	2008	Stage IV extranodal marginal zone B cell lymphoma	1	M	69	R-CHOP	5	therapy	Bilateral patchy GGOs, suggestive for new-onset interstitial pneumonitis. Transbronchial lung biopsy revealed interstitial thickening and type II pneumocyte hyperplasia, consistent with interstitial pneumonitis.	Reversible	Corticosteroids. No lymphoma progression was noted.
Coelho, R.R. et al. [34]	2024	Primary Sjögren’s Syndrome/indolent progressive systemic sarcoid reaction.	1	F	65	Rituximab	Unspecified number over a period of 3 years	9 months after the last cycle	Reticular changes at the basal segments of both lower lobes, enlarged aorto-pulmonary lymph nodes, a small subsolid nodule in the posterior left upper lobe and subpleural micronodules.	Reversible	Rituximab was discontinued; prednisolone (0.5 mg/kg/day) initiated, leading to imaging and functional improvement at 1 month and PET negativity at 4 months; mycophenolate mofetil was proposed as a steroid-sparing agent. PET showed no hypermetabolic activity.
Liu, X. et al. [35]	2008	DLBCL (n = 5) Mantle cell lymphoma (n = 2) Follicular lymphoma (n = 1) B cell lymphoma (n = 1)	9	M-7 F-2	54 (median age)	R-CEOP (n = 8) R-CVP (n = 1)	2 (median value)	9–19 days after the previous infusion of rituximab (median 14 days)	8 patients with bilateral pulmonary diffuse interstitial infiltrations and one patient with unilateral pulmonary flaky interstitial infiltration.	Reversible (8 patients) Fatal (1 patient)	Median steroid duration was 21 days; 8 patients recovered fully, while 1 developed secondary infection and died of respiratory failure 41 days after rituximab-induced pneumonitis. Rituximab was stopped in 4 patients and continued in 4; among those retreated, 2 experienced recurrent pneumonitis.
Wu, Y. et al. [36]	2018	Primary central nervous system lymphoma	1	F	33	R-MAD	5	Two weeks	Bilateral lung fields with diffuse GGOs, suggestive for interstitial pneumonitis	Reversible	High-dose IV steroids; meropenem. Rapid defervescence and marked radiologic improvement within 7 days. Subsequently, the patient received pemetrexed and EA consolidation chemotherapy without ILD relapse, and follow-up imaging showed no lymphoma recurrence.
Rathi, M. et al. [37]	2012	Class IV lupus nephritis with thrombotic microangiopathy	−1	F	26	Rituximab along with plasmapheresis	3	One month	A transbronchial lung biopsy revealed pulmonary fibrosis.	Chronic	IV methylprednisolone pulses and high-dose oral corticosteroids, leading to rapid clinical recovery and normalized oxygenation, though imaging remained unchanged; at 1 month, the patient was asymptomatic.
Liu, C. et al. [13]	2023	B-cell lymphoma	66/831	M-39, F-27		R-CHOP (n = 17) R-CDOP (n = 49) ±prophylactic use of TMP-SMX.			Diffuse pulmonary interstitial infiltrates	Not mentioned	Prophylactic use of TMP-SMX could prevent the occurrence of IP whose risk factor was associated with pegylated liposome doxorubicin after chemotherapy for B-cell lymphoma.
Ban, A.Y. et al. [38]	2021	CD5-negative B-cell lymphoproliferative disorder	−1	F	49	Rituximab, fludarabine, cyclophosphamide	1	Three days	Bilateral GGOs with interlobular septal thickening and arcade-like signs suggestive of OP.	Reversible	IV methylprednisolone to rapid recovery, with ventilatory support discontinued in 1 week and complete HRCT resolution. Rituximab was not resumed due to safety concerns, and obinutuzumab was initiated instead.
Herishanu, Y. et al. [39]	2006	Follicular grade 3 non-Hodgkin lymphoma	−1	M	80	R-CHOP	5	Two days	Progressive subpleural consolidation with GGOs, small cysts, and septal thickening. Biopsy revealed interstitial inflammation, atypical type II pneumocyte hyperplasia, and foamy histiocyte accumulation.	Fatal	IV methylprednisolone (1 mg/kg) was started, but the patient developed progressive respiratory failure and died 10 days after admission.
Sun, Y. et al. [40]	2020	Nongerminal center B cell diffuse large B-cell lymphoma (non-GCB-DLBCL, stage III EB)	−1	M	49	DA-EPOCH-R	5	2 weeks	Diffuse bilateral GGOs. Transbronchial lung biopsy showed thickened alveolar walls, with fibrous tissue hyperplasia and lymphocyte infiltration, suggestive for NSIP.	Reversible	Prednisone (0.8 mg/kg/day) led to symptom resolution and improved oxygenation within 3 weeks; gradual taper was well tolerated. Serial HRCT showed resolution of GGOs, and PFTs improved (FVC 72.5%, DLCO 68.2%).
Ullah, K. et al. [41]	2013	Relapse of non-Hodgkin’s lymphoma	1	M	56	R-CVP	4		Bilateral GGOs, predominantly in the upper-lobes, suggestive for hypersensitivity pneumonitis secondary to rituximab).	Reversible	Rituximab was stopped and high-dose oral steroids tapered over 2 months, leading to complete clinical and radiologic resolution with DLCO improving to 79% predicted.
Arulkumaran, N. et al. [42]	2012	Antineutrophil cytoplasmic antibody-associated vasculitis	−1	M	65	Rituximab and prednisolone	2	15 days	Diffuse GGOs with septal thickening, ill-defined centrilobular nodules, and peripheral consolidations	Reversible	Treatment comprised IV methylprednisolone (3 × 500 mg), cyclophosphamide (2 doses, 2-week interval), rituximab (2 × 1 g, 2 weeks apart), and 7 cycles of plasmapheresis completed before the 2nd rituximab dose. Follow-up CT showed progressive clearance of diffuse abnormalities and resolution of alveolar damage. The patient was discharged from ICU on minimal oxygen and remains in remission on mycophenolate plus tapering prednisolone for ANCA-vasculitis.
Heresi, G.A. et al. [43]	2008	Waldenstrom’s macroglobulinemia	1	M	88	Fludarabine + cyclophosphamide + rituximab	4 doses of rituximab, followed by 4 cycles of fludarabine and cyclophosphamide which were completed 3.5 years before the current presentation, splenectomy, and then, more recently, 8 doses of rituximab (375 mg/m^2^ per administration).	Eight weeks	Bilateral alveolar and interstitial infiltrates; BAL revealed diffuse alveolar hemorrhage; Transbronchial biopsy: interstitial pneumonitis with granulomata.	Reversible	Corticosteroids
Biehn, S.E. et al. [44]	2006	Mucosal-associated lymphoid tissue NHL	1	M	61	Rituximab monotherapy	4	2 months	Metabolically active solid nodular lesions. After levofloxacin, one nodule enlarged with spiculated margins, prompting wedge resection. Histology confirmed BOOP, with no evidence of malignancy.	Reversible	Prednisone 40 mg/day led to rapid improvement in 4 days and complete symptom resolution by 1 month; gradual taper followed, with normalized PFTs at 7 months and minimal residual PET activity.
Alexandrescu, D.T. et al. [45]	2004	Stage IV DLBCL	1	M	65	R-CHOP	1		CT showed new GGOs with near-complete remission of lymphoma; lung biopsy revealed non-necrotic granulomas with mild fibrosis, suggestive for drug-induced hypersensitivity pneumonia.	Fatal	Corticosteroids; CXR showed worsening alveolar infiltrates, suggestive of infection or hemorrhage. Skin biopsy revealed perivascular lymphocytic infiltrate with RBC extravasation, consistent with Schamberg’s disease, a hypersensitivity-related dermatitis. Progression to respiratory failure and death; autopsy showed diffuse alveolar damage with hemorrhage, and cause of death was *S. aureus* sepsis.
Lee, Y. et al. [46]	2006	DLBCL (stage IIIA and IVA)	2	M-2	1. 73 2. 66	R-CEOP	1–7 2–5		1. Subpleural GGO at both lungs—interstitial pneumonitis; 2. Increased centrilobular and subpleural GGO and multiple consolidations in both lung fields—interstitial pneumonitis.	Reversible	Corticotherapy led to symptoms resolution and improvement in lung function in both patients.
Katsuya, H. et al. [47]	2009	DLBCL (n = 6) Follicular lymphoma (n = 2)	8/129	M-2, F-6	60.5 (mean age)	R-CHOP, G-CSF	Not specified		Reticular or GGOs patterns	6 reversible, 1 1 chronic, 1 fatal	Patients who developed *P. jirovecii* infection were also treated with TMP-SMX, together with steroids.
Zhang, X. et al. [48]	2015	DLBCL	1	F	51	R-CHOP	4	Before the 5th cycle	Bilateral GGOs. The lung biopsy revealed areas of pulmonary fibrosis and the presence of inflammatory cells, but was negative for other pathogens	Reversible	IV methylprednisolone (1 mg/kg/day) and oxygen led to rapid recovery with CT and TNF-α normalization in 2 weeks. She received C-VED chemotherapy, and rituximab was discontinued.
Ghesquieres, H. [49]	2005	DLBCL	2	M-2	1–52 2–59	R-ACVBP	1–3 2–4	1. 12 days 2. 10 days	1. Chest radiography revealed slight bilateral basal pulmonary infiltrate and CT scan confirmed the presence of bilateral ground-glass opacities. 2. Left pneumothorax and diffuse bilateral confluent infiltrates.	1. reversible; 2. fatal	1. High-dose steroids led to rapid clinical and radiologic recovery; subsequent cycles omitted rituximab/bleomycin, and steroids were stopped after 45 days without relapse; 2. Despite methylprednisolone (1 mg/kg) initiated 21 days after symptom onset, the patient progressed to respiratory failure and died in the ICU.
Nakamura, K. et al. [50]	2016	Systemic sclerosis	1	F	70	Rituximab, cyclosporin, angiotensin-converting enzyme inhibitors	Not specified	One month after the first rituximab infusion	Bilateral GGO	Fatal	Pulse methylprednisolone and broad-spectrum antibiotics were given, but the patient died of respiratory failure 20h after hemoptysis; postmortem CT showed diffuse bilateral infiltrates.
Macartney, C. et al. [51]	2005	DLBCL	1	M	52	R-CHOP, granulocyte colony stimulating factor, pegylated filgrastim	4	14 days	Diffuse bilateral GGOs with patchy opacities; transbronchial biopsy revealed BOOP with fibroblastic proliferation and foamy macrophages.	Reversible	Prednisolone produced rapid radiologic and functional improvement. Two further CHOP cycles without rituximab/filgrastim were uneventful, and the patient remains in complete remission 9 months post-therapy.

M—male; F—female; ILD—interstitial lung disease; DLBCL—diffuse large B-cell lymphoma; R-CHOP—Rituximab combined CHOP-like regimen; CHOP—cyclophosphamide, doxorubicin, vincristine, prednisolone; GGO—ground glass opacities; FL—follicular lymphoma; IV—intravenous; HRCT—high resolution computer tomography; NSIP—nonspecific interstitial pneumonia; DLCO—diffusing capacity of the lungs for carbon monoxide; BAL—bronchoalveolar lavage; PCR—polymerase chain reaction; CT—computer tomography; ICU—intensive care unit; R-ILD—Rituximab-induced interstitial lung disease; anti-TNF—anti-tumor necrosis factor; BOOP—Bronchiolitis Obliterans Organizing Pneumonia (BOOP); NHL—non-Hodgkin lymphoma; PET—positron emission tomography; R-CDOP—rituximab + cyclophosphamide + liposomal doxorubicin (D) + vincristine (O) + prednisone; R-CEOP—rituximab + cyclophosphamide + etoposide (E) + vincristine (O) + prednisone; R-CEV—rituximab + cyclophosphamide + etoposide + vincristine; R-CVP—rituximab + cyclophosphamide + vincristine + Prednisone; R-MAD—rituximab + mitoxantrone + cytarabine (Ara-C) + dexamethasone; EA—etoposide + cytarabine; TMP-SMX—trimethoprim–sulfamethoxazole; OP—organizing pneumonia; G-CSF—granulocyte colony-stimulating factor; DA-EPOCH-R—dose-adjusted etoposide, prednisone, vincristine (oncovin), cyclophosphamide, doxorubicin (hydroxydaunorubicin), plus rituximab; PFT—pulmonary function test; FVC—forced vital capacity; ANCA—anti-neutrophil cytoplasmic antibodies, CXR—chest radiography; R-ACVBP = rituximab + doxorubicin (Adriamycin) + cyclophosphamide + vindesine + bleomycin + prednisone.

## 4. Discussion

Rituximab, known as the first therapeutic mouse–human chimeric monoclonal antibody introduced in cancer treatment, targets CD20, a surface glycoprotein expressed on both malignant and normal B cells; when combined with chemotherapy regimens like CHOP, rituximab has achieved curative outcomes even in advanced cases of lymphoma. Since its approval in 1997 for the treatment of NHL, this chimeric anti-CD20 antibody has seen its therapeutic indications broadened beyond malignant diseases, as it was proved effective for some severe immune diseases [52,53], including rheumatologic and connective tissue disorders (ANCA-associated vasculitis, rheumatoid arthritis, systemic sclerosis, Behçet’s disease), hematologic autoimmune conditions (autoimmune hemolytic anemia, immune thrombocytopenia, cryoglobulinemia, Castleman’s disease), dermatologic diseases (pemphigus vulgaris, bullous pemphigoid), neurological disorders (myasthenia gravis, neuromyelitis optica spectrum disorder) and renal involvement (nephrotic syndrome, Goodpasture’s disease, lupus nephritis) [54]. 

Our systematic research found that most R-ILD-relevant articles were single case reports (n = 30), primarily describing B-cell non-Hodgkin lymphomas such as DLBCL, follicular, marginal zone, and Mantle cell subtypes, along with rarer entities including Waldenström’s macroglobulinemia, primary central nervous system lymphoma and others. A smaller subset reported autoimmune or immune-mediated diseases, such as idiopathic thrombocytopenic purpura, systemic lupus erythematosus with lupus nephritis, neuromyelitis optica, pemphigus vulgaris, fibrillary glomerulonephritis, ANCA-associated vasculitis, primary Sjögren’s syndrome, and systemic sclerosis. In addition, case series (n = 5) and cohort studies (n = 5) provided data on larger groups, again predominantly with B-cell NHL. Case series typically reported small numbers of DLBCL, follicular, Mantle cell, and small B-cell lymphomas, including relapsed disease. Cohort studies described broader populations, with the largest series including 256 DLBCL cases and smaller groups of marginal zone, follicular, mantle cell, Burkitt, high-grade B-cell lymphomas and leukemia. Overall, B-cell NHL accounted for the vast majority of cases, while autoimmune diseases represented a smaller but significant fraction.

The incidence of rituximab-induced interstitial pneumonia is not systematically reported and various articles make use of the term ‘rare’ to qualitatively characterize its incidence. There were six cohort studies identified [13,14,15,32,47,55] which reported the incidence of R-ILD among rituximab-exposed patients. Based on this data, we estimated a global incidence of approximately 14% in lymphoma patients treated with various rituximab-containing regimens. This suggests that R-ILD may occur more frequently than typically reported. However, this finding should be interpreted with caution, as some minor radiological abnormalities attributed to rituximab might not truly represent R-ILD, and the available data refers exclusively to lymphoma patients undergoing complex therapeutic protocols that can themselves induce pulmonary toxicity. Still rituximab-containing regimens seem to be linked to a higher incidence of interstitial disease: CHOP vs. RCHOP and CDOP vs. RCDOP 0, 1.8, 17.4 and 21.1% [55].

Time from exposure to onset of disease is a particularly important parameter as it may prove useful in clinical practice; there were 49 individual cases where this information could be retrieved. RILD was reported from the first dose to the 8th treatment cycle; time intervals varied from one week after the first exposure to nine months after the last dose. There is published data on the safety of rituximab for rheumatological conditions suggesting 4 cycles and 15 days following the last administration as high risk moments; the values aggregated from the case reports (39 lymphoma patients and 9 rheumatological/dysimmune conditions) are similar, but support a more cautious approach to patient supervision [56].

There is limited data on the clinical features of R-ILD given the low specificity and sensitivity of cardinal respiratory signs and symptoms. Using the data available extracted from the 49 individual cases, we estimated that among R-ILD patients, dyspnea is the most prevalent respiratory symptom (73%), followed by dry cough (61%) and fever (47%); it is worth noting that roughly 10% of the cases were asymptomatic. These figures are similar to previously published data [56]; the presence of such clinical features among rituximab-receiving patients should prompt additional testing while also considering the underlying conditions and their potential complications.

The diagnosis of any interstitial lung disease is typically imagistics-based—such results were available for all 49 cases, generally in the form of computed tomography. Ground glass opacities were the most prevalent pulmonary lesion (reported in 24 cases accounting for 49% of total); other patterns included larger bilateral infiltrative lesions (15 cases accounting for 30% of total), consolidation (5 cases, 10%) and rarer, atelectasis, multifocal pneumonia, cryptogenic pneumonia or pneumothorax. We found no evident associations between the interstitial pattern and the underlying condition or clinical features.

Pathology data was limited: a non-specific interstitial pneumonia pattern was reported in four cases and various other findings were spuriously mentioned—hyaline membranes, poorly formed granulomas, interstitial pneumonia; such findings might hint at different toxicity mechanisms.

Lung function testing is mentioned only for 21 cases showing restrictive patterns for 18 patients, obstructive for one case, and 2 mixed defects. Transfer capacity was assessed for 15 cases; only one patient presented with normal transfer parameters (asymptomatic and showing only reticulations). Although data is limited, transfer capacity seems reliable in detecting R-ILD at least when chronic forms are suspected but it seems underutilized, possibly because of limited availability. 

The outcome of R-ILD was variable: 30 cases (61%) recovered completely, 10 cases (20%) showed improvement and 9 cases (19%) concluded in death. Lethal outcomes occurred in 7 lymphoma cases and two non-malignant, rheumatologic conditions.

In the malignant forementioned conditions, rituximab is integrated into multiple agent chemotherapy regimens, most frequent CHOP-based protocols, as well as bendamustine-containing schedules for indolent lymphomas. Available studies suggest that combination regimens are more frequently associated with the development of interstitial lung disease compared to rituximab monotherapy, underscoring the need for vigilant monitoring of pulmonary function during their administration [57,58]. On the other hand, when rituximab is used for systemic immune-mediated diseases, there seems to be more variability and flexibility: monotherapy, maintenance regimens, combination strategies with corticosteroids, plasmapheresis or other immunosuppressive agents; this difference makes comparisons difficult. Still, minor adverse events were reported more frequently among patients with lymphoma compared to those with immune-mediated disorders, with infectious complications representing the predominant category. Even though such events have been observed, rituximab continues to be regarded as a safe and generally well-tolerated therapeutic agent when used appropriately [59].

The major challenge lies in achieving an accurate and timely diagnosis of R-ILD, both in malignant and autoimmune disorders. Clinical features such as dyspnea, cough or fever may be ascribed to the underlying condition or various intercurrent infections; computed tomography is not always readily available, whereas raised concerns linked to radiation exposure and ground glass lesions are considered a non-specific finding. A definite R-ILD diagnosis is an extensive exclusion process; therefore, a multidisciplinary approach should be systematically considered in patients on either rituximab monotherapy or combination regimens when a reasonable suspicion arises [60]. 

**Figure 3:**
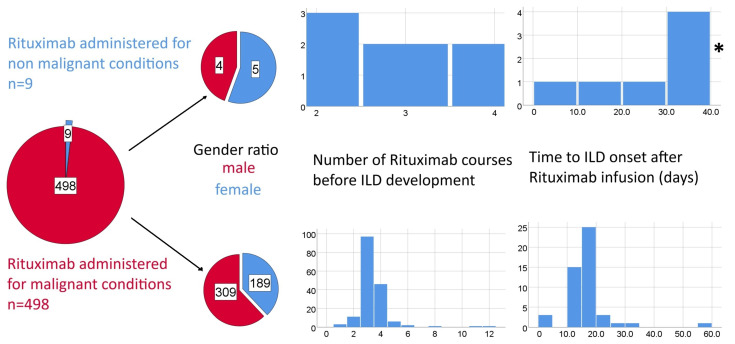
Potential differences between Rituximab-induced ILD associated with malignant and non-malignant conditions in terms of gender ratios expressed as pie charts and time to ILD (number of therapeutic cycles and time from last rituximab perfusion) expressed as histograms. * one outlier value of 270 days was excluded from the histogram due to scaling issues.

**Figure 4:**
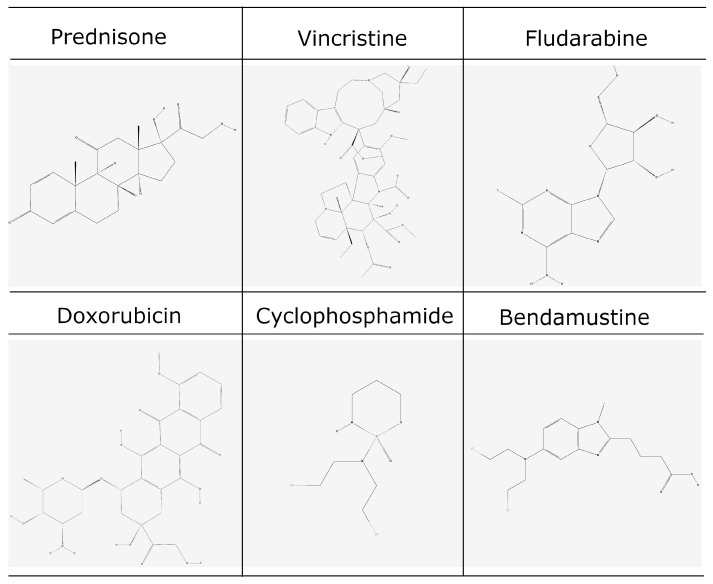
Chemical structures of frequently used agents in lymphoma therapeutic protocols (adapted from PubChem).

The development of ILD following rituximab shows significant timing heterogeneity: ranging from a few days after the first infusion to several months after treatment completion; the majority of the collected cases occurred within the first two to five treatment cycles, with cohort data indicating a median onset around the third cycle. R-ILD tends to manifest earlier in patients with autoimmune disorders treated with rituximab as monotherapy (Figure 3), compared to those receiving multi-agent chemotherapy for lymphoma, suggesting a potential cumulative effect of combination regimens in the latter group as many agents have known pulmonary toxicities (Figure 4).

The majority of R-ILD patients presented with GGOs as the main imagistic anomaly—mainly bilateral and diffuse, occasionally accompanied in some cases by reticulations, nodules or consolidations. There were also patterns suggestive for NSIP, UIP, organizing pneumonia or hypersensitivity pneumonitis. In several cases, transbronchial biopsies were performed and the confirmation was performed through histopathological examinations, revealing interstitial pneumonitis, fibrotic changes and in severe cases even alveolar hemorrhage. These findings may be considered a potential tool to predict the evolution of R-ILD. In the analyzed studies, the GGOs were most often reversible following the corticosteroid therapy, in some cases even without the discontinuation of rituximab, but with additional support, such as TMP-SMX prophylaxis. Corticosteroids, most commonly prednisone or methylprednisolone, represented the main therapy for R-ILD, leading to favorable outcomes for most cases, as evidenced by improvements in pulmonary function and CT scans. There is no consensus on specific corticoid agent, optimum dose or therapy duration; other immunomodulatory agents are postulated as potentially useful. Such immune modulation might be not always necessary: e.g., for one patient with NMOSD-AQP4, discontinuation of rituximab alone achieved complete clinical and functional recovery [20]. Major pulmonary involvement, manifesting as NSIP or BOOP/COP, frequently failed to achieve full function restoration under treatment, evolving toward chronic disease; patients with fibrotic changes had a similar evolution and permanent rituximab discontinuation was usually necessary. Fatal outcomes were associated with acute onset and extensive diffuse lung injury, as well as severe alveolar hemorrhages, which led despite the maximal therapy to respiratory failure and death. Regarding gender distribution, outcomes appeared heterogeneous; however, chronic evolution seemed to occur more often in male patients with advanced fibrotic damage. 

As discontinuation of rituximab may, in certain cases, exacerbate the underlying disease, restarting after the ILD resolution is reported as possible but there is no consensus on how the rechallenge should be performed or if it is necessary; ILD recidive after restarting rituximab is also reported. Along this line it is important to highlight that within the largest cohort study from our analysis (321 DLBCL patients) almost 88% of the subjects were suitable to resume Rituximab therapy following successful management of R-ILD [14]. Limited case series data supports restarting rituximab, albeit prudence is recommended if some risk predictors are present such as age over 70, poor ECOG score or recent surgery [61]. This decision is therefore complex and should ideally be made within a multidisciplinary team, while weighing the risks of pulmonary toxicity against the benefits of ongoing disease control.

When managing R-ILD, it should be noted that underlying conditions such as lymphomas or connective tissue disorders have the potential of causing or being associated with various pulmonary phenomena not necessarily related to rituximab. Even more rituximab may also cause pulmonary events by multiple mechanisms—infections are frequently reported. Along these lines, attempts to develop clinical tools able to differentiate between these mechanisms as therapeutic options are different [8]. The lung toxicity mechanism of rituximab is not clear—the NRLP3 IL-1β and IL-18 pathway may play a role at least for some patients [19].

Subclinical, low-intensity infectious processes may also be involved; data from a cohort study (n = 1127 lymphoma patients of whom 321 developed pulmonary toxicity) which included bronchioloalveolar lavage supports the involvement of various microorganisms, mainly viral agents, but also *Pneumocystis jirovecii*, fungi and Mycobacterium tuberculosis. Furthermore, the authors postulate a link between the dominant cellular population in the lavage and the nature of the pulmonary lesion (neutrophilic versus lymphocytic) although the connection is not clearly substantiated or explored [8]. In another relevant cohort study, Liu et al. demonstrated the importance of using trimethoprim-sulfamethoxazole in preventing the development of interstitial pneumonitis with *Pneumocystis jirovecii* in patients with NHL. When patients were classified into two groups—those receiving prophylactic antibiotics and those without prophylaxis during chemotherapy combined with rituximab—it was observed that no cases of interstitial pneumonitis occurred in the prophylaxis group, whereas an incidence of 9.4% was reported in the non-prophylaxis group [13].

## 5. Conclusions

R-ILD represents an important potential adverse effect in both malignant and autoimmune conditions treated with rituximab, either as monotherapy or in combination with chemotherapy. Its incidence is probably higher than previous estimates as clinical manifestations are frequently nonspecific, making timely recognition challenging. Ground glass opacities are the most prevalent computed tomography pattern associated with R-ILD. A multidisciplinary approach is essential, with an emphasis on early diagnosis and rapid initiation of therapy. Corticosteroid treatment leads to resolution in most cases, yet clinicians should remain aware that severe presentations may progress to respiratory failure and fatal outcomes.

## Data Availability

All information provided in this review is supported by the relevant references.

## Supplementary Materials

The following supporting information can be downloaded at: https://www.mdpi.com/article/10.3390/cancers17233786/s1, File S1: PRISMA 2020 Checklist [62].
